# The Complex Nature of Bilinguals' Language Usage Modulates Task-Switching Outcomes

**DOI:** 10.3389/fpsyg.2016.00560

**Published:** 2016-04-25

**Authors:** Hwajin Yang, Andree Hartanto, Sujin Yang

**Affiliations:** ^1^School of Social Sciences, Singapore Management UniversitySingapore, Singapore; ^2^Department of Psychology, Ewha Womans UniversitySeoul, South Korea

**Keywords:** bilingualism, task switching, the adaptive control hypothesis, shifting EF, switch costs, mixing costs

## Abstract

In view of inconsistent findings regarding bilingual advantages in executive functions (EF), we reviewed the literature to determine whether bilinguals' different language usage causes measureable changes in the shifting aspects of EF. By drawing on the theoretical framework of the adaptive control hypothesis—which postulates a critical link between bilinguals' varying demands on language control and adaptive cognitive control (Green and Abutalebi, 2013), we examined three factors that characterize bilinguals' language-switching experience: (a) the interactional context of conversational exchanges, (b) frequency of language switching, and (c) typology of code-switching. We also examined whether methodological variations in previous task-switching studies modulate task-specific demands on control processing and lead to inconsistencies in the literature. Our review demonstrates that not only methodological rigor but also a more finely grained, theory-based approach will be required to understand the cognitive consequences of bilinguals' varied linguistic practices in shifting EF.

## Introduction

Executive functions (EF) refer to a multifaceted construct of a general control process that consists of three postulated functions: *inhibition, updating*, and *shifting* (Miyake et al., 2000). Recent studies of the cognitive advantages of bilingualism in EF report inconsistent findings, and contend that bilingual advantages in EF do not exist—and, further, that there is no compelling evidence that any specific bilingual experience stimulates a certain aspect of EF (e.g., Paap et al., 2015, p. 272). This claim, however, requires a more rigorous, theory-driven approach to identify potential moderators or confounds that contribute to the presence (or absence) of bilingual advantages in EF (Yang et al., 2016).

In this review, we focus on the importance of bilinguals' varied language-switching experiences in modulating shifting aspects of EF, and suggest that specific bilingual experiences can potentially moderate bilingual advantages in EF. We examined three aspects of bilinguals' language-switching experiences: (a) the interactional context of conversational exchanges, (b) language-switching frequency, and (c) typology of code-switching, which refers to the alternation between two or more languages. Moreover, given that varying task demands modulate bilingual advantages in EF (Costa et al., 2009; Yang and Yang, 2016), we also examined whether divergent findings in the literature can be attributed to task-specific demands driven by methodological differences or other factors, such as age.

## Inconsistencies in the literature

Shifting EF has typically been assessed by the task-switching paradigm (e.g., color-shape and number-letter tasks), in which participants are prompted to alternate between two different tasks. The paradigm typically derives two task-switching costs that have been found to implicate different control mechanisms (Braver et al., 2003; Rubin and Meiran, 2005). Switch costs refer to slower responses on task-switch trials than on non-switch trials, and thereby reflect the actual cost of switching between different task sets. Switch costs are driven by local control mechanisms that involve (a) task-set reconfiguration, which is the ability to change from one task set to another (Rogers and Monsell, 1995; Meiran, 1996), and (b) inhibition of proactive interference, which is the ability to regulate interference from the previously formed task set (Wylie and Allport, 2000; Philipp et al., 2008). Mixing costs, on the other hand, refer to slower responses on task-repeat trials in mixed-task blocks than in pure blocks—which consist of a single task—and thereby reflect the cost of monitoring and coordinating multiple streams of incoming information. Mixing costs entail the activation of global control mechanisms that are necessary to monitor task cues, maintain two competing tasks/response sets, and make task-appropriate decisions (Braver et al., 2003; Rubin and Meiran, 2005).

Bilinguals' language switching requires the ability to reconfigure language sets while simultaneously inhibiting potential interference from a previously relevant, but now irrelevant, language. This has led some to argue that bilingual advantages likely reside in switch costs (Garbin et al., 2010; Prior and MacWhinney, 2010; Prior and Gollan, 2011; Houtzager et al., 2015). On the other hand, since bilinguals constantly monitor two languages that are activated simultaneously, others have argued that bilingual advantages should be evident in mixing costs (e.g., Barac and Bialystok, 2012; Gold et al., 2013; Wiseheart et al., 2016). While the two camps debate whether bilingualism benefits either switch costs or mixing costs, still other researchers have failed to replicate bilingual advantages in either context, and therefore argue that bilingualism does not benefit shifting-specific EF (Hernández et al., 2013; Paap and Greenberg, 2013; Paap and Sawi, 2014; Mor et al., 2015).

## Interactional context of conversational exchanges

Recently, Green and Abutalebi (2013) proposed the adaptive control hypothesis, which holds that the different interactional contexts of bilinguals' conversational exchanges place varying demands on language control, which in turn adaptively alter their cognitive-control capacities. Specifically, Green and Abutalebi distinguish three different interactional contexts: (a) the *dual-language* context, in which bilinguals use two languages (L1 and L2) within the same context (e.g., at home and work); (b) the *single-language* context, in which bilinguals speak only one language in one environment, and therefore rarely switch languages (e.g., L1 at home and L2 at work); and (c) the *dense code-switching* context, in which bilinguals routinely mix the linguistic elements (e.g., words) of two languages within a single utterance (i.e., intrasentential code-switching). For instance, an English-Tagalog bilingual might say, “Wala akong cash pang grocery ngayon”; *I do not have cash for grocery today* (Green and Abutalebi, 2013, p. 518).

The adaptive control hypothesis suggests that bilinguals' interactional context is a key factor that modulates cognitive advantages in EF. Specifically, bilinguals' dual-language context engages a more complex and taxing level of control processes of goal maintenance, conflict monitoring, and interference suppression, and therefore facilitates more adaptive cognitive control than either the single-language or dense code-switching context. Given that bilinguals in a dual-language context should experience not only more frequent but also more qualitatively challenging language switching, it is conceivable that the dual-language context will promote shifting EF—and therefore enhance task-switching performance—while the single-language and dense code-switching contexts will not. Consistently, Hartanto and Yang (2016) found that bilinguals who mainly engage in a dual-language context showed smaller switch costs than those who mainly engage in a single-language context. Moreover, Guerrero et al. (2015) found that bilingual children who spoke a more balanced mixture of languages at home (e.g., 50% English and 50% another language) displayed lower switching costs in a trail-making task than those who spoke only one language at home. Verhagen et al. (2015) also reported that bilingual children whose parents spoke different languages performed significantly better on the Stroop and delay-of-gratification tasks than bilinguals whose parents spoke the same language as the child. It is clear, therefore, that the interactional context of bilinguals' conversational exchanges is a critical factor that renders bilingual experience more advantageous in shifting EF and, in turn, enhances task-switching performance.

## Frequency of language switching

Because bilinguals' frequent language switching likely requires greater control of each language, it is reasonable to expect that bilinguals who frequently switch languages will have enhanced task-switching skills. Prior and Gollan (2011) first examined this issue by comparing monolinguals to Spanish-English and Mandarin-English bilinguals who differed in their self-reported frequency of language switching. Not surprisingly, they found that only Spanish-English bilinguals—who were regarded as frequent language switchers—had smaller switch costs than monolinguals, suggesting that there may be a minimum threshold for language-switching frequency that confers advantages on task switching.

Language-switching frequency has also been found to modulate inhibitory control, which is closely related to task switching (Friedman et al., 2006; Miyake and Friedman, 2012). Specifically, Verreyt et al. (2016) compared three groups of bilinguals—unbalanced bilinguals, balanced non-switching bilinguals, and balanced switching bilinguals—on flanker and Simon tasks. They found that of the three groups, balanced switching bilinguals had the significantly lowest flanker and Simon effects. More importantly, frequency of switching was negatively correlated with both flanker and Simon effects, which suggests that language-switching frequency is important for efficient inhibitory control. In the same vein, Woumans et al. (2015) found that higher language-switching proficiency is associated with lower congruency effects in Simon and attention-network tasks.

In contrast, Paap and Greenberg (2013) did not find that frequent language switching confers benefits on either task-switching costs or inhibitory control. Given this discrepancy, it is notable that they employed the percentage of daily English usage (L1) as a proxy measure of language-switching frequency; they reasoned that the smaller percentage of L1 usage reflects considerable usage of the other language (L2), and therefore can approximate the frequency of language switching between L1 and L2. Albeit plausible, the validity of this approximation may be questionable, since the percent usage of L1 does not always indicate the frequency of language switching. That is, 50% of L1 use can be achieved without language switching by speaking L1 exclusively at home and L2 at work (i.e., a single-language context).

Not surprisingly, other studies have found no correspondence between the percent usage of L1 relative to L2 and self-reported language-switching frequency. For example, Prior and Gollan's (2011) Spanish-English and Mandarin-English bilinguals used English at comparable percentages (i.e., *M*_*S*−*E*_ = 84.6%, *M*_*M*−*E*_ = 86.6%), but their language-switching frequency differed significantly (*M*_*S*−*E*_ = 3.2, *M*_*M*−*E*_ = 2.4 on a 5-point Likert scale, with 1 = *almost never* and 5 = *constantly*). Likewise, Paap and Sawi (2014) tested bilinguals who reported speaking L1 about 70% of the time, but their report of actual switching frequency was limited to only a couple of times a day. Given this, findings based on the percent usage of L1 (or L2) as a proxy measure of language-switching frequency should be interpreted with caution.

## The typology of code-switching

The adaptive control hypothesis proposes that the challenging quality of bilinguals' linguistic practices plays a key role in triggering more adaptive cognitive control (Green and Abutalebi, 2013). In support of this hypothesis, Macnamara and Conway (2013) found that bilinguals' cognitive control and working memory were positively influenced by the degree of bilingual management demands—the extent to which bilinguals engage cognitive control to appropriately use the two languages—and the amount of experience managing the bilingual demands. Therefore, we examined the influence of different types of code-switching on shifting EF.

Broadly, bilinguals' language switching is classified as either intrasentential code-switching (i.e., mixing linguistic units from two languages within a sentence) or intersentential code-switching (i.e., interchanging two or more languages between sentences; e.g., Brice, 2000). Intrasentential code-switching is characterized by loan words that are integrated into the other language's syntactic context (i.e., grammar). For instance, a Singapore bilingual might say, “I like to *makan* some good seafood”; *makan* means “eat” in Malay. Even when the bilingual does not understand Malay grammar, the speaker is still able to switch languages easily, because intrasentential code-switching does not necessarily entail a substantial shift from one linguistic set to another. Intrasentential code-switching, therefore, facilitates language production by permitting the speaker to use whatever comes most readily (e.g., Gollan and Ferreira, 2009; Green and Abutalebi, 2013). In line with this, Soveri et al. (2011) found that frequency of intrasentential code-switching showed a negative correlation with (i.e., attenuated) mixing costs, but a trend of positive correlation with (i.e., increased) switch costs, suggesting that intrasentential code switching may not benefit switch costs.

On the other hand, intersentential code-switching is believed to be more demanding than intrasentential code-switching, as the former requires language-set reconfiguration and proactive inhibition. Therefore, more frequent intersentential code-switching likely facilitates bilingual advantages in task switching. In support of this view, recent studies suggest that the demands of language control modulate task-switching performance. For instance, Hartanto and Yang (2016) identified a trend in which, when bilinguals' SES and intelligence were controlled for, intersentential code-switching negatively predicted switch costs (i.e., reduced switch costs), while intrasentential code-switching positively predicted switch costs (e.g., increased switch costs) in the same regression model.

Relatedly, it seems that cognitive demand for intersentential code-switching is further influenced by who initiates the discourse—i.e., self vs. other, which is sometimes referred to as voluntary vs. involuntary (e.g., Gollan et al., 2014). Self-initiated intersentential code-switching seems to be less costly and relatively less taxing than other-initiated intersentential code-switching, because the former permits sufficient preparation time to reconfigure language sets, while other-initiated intersentential code-switching is triggered unexpectedly and therefore imposes greater demands on language-set reconfiguration and proactive inhibition. As demonstrated by Gollan et al. (2014), self-initiated voluntary switching is associated with significantly lower language-switching costs than is the case with other-initiated switching. This notion is consistent with the adaptive control hypothesis, which proposes that language switching in response to an interchange with another speaker incurs greater costs than voluntary language switching (Green and Abutalebi, 2013). Accordingly, more frequent exercise of other-initiated intersentential code-switching is likely to be more beneficial for switch costs than self-initiated intersentential code-switching. We suggest, therefore, that various factors may modulate the impact of complex intersentential code-switching on task-switching performance. To date, however, this issue has received little attention, and warrants more in-depth research. In sum, the typology of code-switching is an important factor that sheds light on the demands of various bilingual experiences and their impact on task-switching performance.

## Variations in the task-switching paradigm

In view of the variety of EF tasks being used to test bilingual effects, inconsistent conclusions regarding bilingual effects on EF can be attributed to task-specific demands driven by methodological differences. Not surprisingly, findings in the literature demonstrate that even minor parametric variations can modulate bilingual advantages in EF through diverse levels of task-specific demands (Costa et al., 2009; Yang and Yang, 2016). Little is known, however, about whether this is the case in relation to shifting EF, which is assessed by a task-switching paradigm. We reviewed the literature, therefore, to examine whether the discrepant findings of bilingual effects on shifting EF can be attributed to varying task demands in task switching. To maintain a reasonable scope, we narrowed our focus within the task-switching paradigm (see Table 1; Monsell, 2003) and excluded variants that have been known to implicate other aspects of EF, despite their ability to derive task-switching costs (e.g., flanker and Simon tasks; e.g., Paap and Sawi, 2014).

**Table 1:** **Summary of studies examining bilingual benefit using a task-switching paradigm (task-cuing paradigm) in adults**.

**Study**	**Participants**	**Mean age**	**Number of trials**	**Cue**	**Response mapping^a^**	**RC^b^**	**Task-switching outcome**
Garbin et al., 2010	Bilinguals (*N* = 19) Monolinguals (*N* = 21)	Young adult (20.9)	60 trials (30 mixed-switch and 30 mixed-repeat)	Verbal	overlapping	50%	Switch-cost advantages in bilinguals
Prior and MacWhinney, 2010	Bilinguals (*N* = 47) Monolinguals (*N* = 45)	Young adult (19.1)	288 trials (144 pure-repeat, 72 mixed-switch and 72 mixed-repeat) sandwich design	Non-verbal	non-overlapping	100%	Switch-cost advantages in bilinguals
Prior and Gollan, 2011	Spanish-English bilinguals (*N* = 41) Mandarin-English bilinguals (*N* = 43) Monolinguals (*N* = 47)	Young adult (19.9)	288 trials (144 pure-repeat, 72 mixed-switch and 72 mixed-repeat)	Non-verbal	non-overlapping	100%	Relative switch-cost advantages after controlling for parents' educational level in Spanish-English bilinguals, but not in Chinese-English bilinguals
Soveri et al., 2011	Finnish-Swedish bilinguals (*N* = 38)	Older adult (52.8)	144 trials (64 pure-repeat, 32 mixed-switch, and 48 mixed-repeat)	Non-verbal	overlapping	50%	Mixing-cost advantages in high language switching bilinguals
Hernández et al., 2013; experiment 3	Bilinguals (*N* = 38) Monolinguals (*N* = 39)	Young adult (19.9)	288 trials (144 pure-repeat, 72 mixed-switch and 72 mixed-repeat)	Non-verbal	non-overlapping	100%	No bilingual advantages
Paap and Greenberg, 2013	Bilinguals (*N* = 109) Monolinguals (*N* = 144)	Young adult (NA)	288 trials (144 pure-repeat, 72 mixed-switch and 72 mixed-repeat)	Non-verbal	non-overlapping	100%	No bilingual advantages
Gold et al., 2013; experiment 1	Older adult bilinguals (*N* = 15) Older adult monolinguals (*N* = 15)	Older adult (63.7)	240 trials (80 pure-repeat, 80 mixed-switch, 80 mixed-repeat)	Verbal	overlapping	50%	Mixing-cost advantages in bilinguals
Gold et al., 2013; experiment 2	Older adult bilinguals (*N* = 20) Younger adult bilinguals (*N* = 20) Older adult monolinguals (*N* = 20) Young adult bilinguals (*N* = 20)	Young adult (31.9) and Older adult (64.2)	240 trials (80 pure-repeat, 80 mixed-switch, 80 mixed-repeat)	Verbal	overlapping	50%	No bilingual advantages (*p* = 0.056 trend toward bilingual advantages in mixing costs in older adults)
Paap and Sawi, 2014	Bilinguals (*N* = 58) Monolinguals (*N* = 62)	Young adult (24.6)	288 trials (144 pure-repeat, 72 mixed-switch and 72 mixed-repeat)	Non-verbal	non-overlapping	100%	No bilingual advantages
Wiseheart et al., 2016	Bilinguals (*N* = 31) Monolinguals (*N* = 37)	Young adult (19.1)	150 trials (50 pure-repeat, 50 mixed-switch and 50 mixed-repeat)	Non-verbal	overlapping	0%	Mixing-cost advantages in bilinguals
Mor et al., 2015	Bilinguals with ADHD (*N* = 20) Bilinguals control (*N* = 20) Monolingual with ADHD (*N* = 20) Monolingual control (*N* = 20)	Young adult (24.6)	288 trials (144 pure-repeat, 72 mixed-switch and 72 mixed-repeat) sandwich design	Non-verbal	non-overlapping	100%	No bilingual advantages
Qu et al., 2015; ScAc condition^c^	Chinese-English bilinguals (*N* = 32) Chinese> monolinguals (*N* = 32)	Young adult (21.1)	118 trials (40 pure-repeat, 10 mixed-switch, and 68 mixed-repeat)	Verbal	overlapping	0%	Bilingual advantages in switch costs and monolingual advantages in mixing costs
Qu et al., 2015; ScAc condition^c^	Chinese-English bilinguals (*N* = 32) Chinese> monolinguals (*N* = 32)	Young adult (21.1)	118 trials (40 pure-repeat, 10 mixed-switch, and 68 mixed-repeat)	Verbal	overlapping	0%	Bilingual advantages in switch costs
Qu et al., 2015; ScAc condition^c^	Chinese-English bilinguals (*N* = 32) Chinese monolinguals (*N* = 32)	Young adult (21.1)	118 trials (40 pure-repeat, 10 mixed-switch, and 68 mixed-repeat)	Verbal	overlapping	0%	Bilingual advantages in switch costs
Qu et al., 2015; ScAc condition^c^	Chinese-English bilinguals (*N* = 32) Chinese monolinguals (*N* = 32)	Young adult (21.1)	118 trials (40 pure-repeat, 10 mixed-switch, and 68 mixed-repeat)	Verbal	overlapping	0%	No bilingual advantages
Houtzager et al., 2015	Dutch-Frisian bilinguals (*N* = 50) German bilinguals (*N* = 50)	Older adult (60.2)	192 trials (96 pure-repeat, 48 mixed-switch, and 48 mixed-repeat)	Non-verbal	non-overlapping	100%	Bilingual advantages in switch costs

CSI, cue-to-stimulus interval; RCI, response-to-cue interval; RC, response compatibility; SCL, single-language context; DCL, dual-language context.

a Overlapping response mapping occurs when each response key is assigned to two responses (e.g., “green” and “triangle”) on the color vs. shape tasks, while non-overlapping response mapping occurs when each response key is assigned to only one response (e.g., “green”).

b RC (response compatibility) indicates the proportion of trials in which the stimulus and response are compatible in the color-shape switching task. For instance, on compatible trials, the bivalent stimulus (e.g., “green triangle”) correctly matches the response associated with “green” and “triangle.”

c Qu et al. (2015) manipulated the cognitive demands of suppression and activation across 4 different task-switching tasks. ScAc, suppress one set of conflicting responses while simultaneously activating another set of conflicting responses; ScAϵ, suppress one set of conflicting responses while simultaneously activating another set of non-conflicting responses; SϵAc, suppress one set of non-conflicting responses while simultaneously activating another set of conflicting responses; SϵAϵ, suppress one set of non-conflicting responses while simultaneously activating another set of non-conflicting responses.

### Varying task demands

It has been documented that procedural changes in color-shape switching tasks substantially affect task-switching performance in bilinguals due to varying task demand (i.e., difficulty; Kiesel et al., 2010; Vandierendonck et al., 2010). Notably, the type of response mapping—i.e., the arrangement of response keys for two different (color and shape) tasks—has yielded mixed results. Studies with non-overlapping response-stimulus sets—i.e., separate response keys are mapped to different answers for each of the two task sets—failed to find bilingual advantages in mixing costs (e.g., Prior and Gollan, 2011; Hernández et al., 2013; Paap and Greenberg, 2013), while those with overlapping response-stimulus sets—i.e., the same response keys are (re)mapped to each of the two tasks—reported bilingual advantages in mixing costs (e.g., Soveri et al., 2011; Gold et al., 2013; Qu et al., 2015; Wiseheart et al., 2016). One possible reason for this discrepancy is that tasks with overlapping response mapping are more demanding, and therefore more sensitive to detect bilingual advantages in mixing costs (Mayr, 2001). According to the task-switching literature, overlapping response mapping increases task interference by triggering greater response competition associated with tasks (Gade and Koch, 2007). Therefore, heightened demand for task competition—which is apparent in overlapping response mapping—likely enhances task sensitivity to capture participants' variations in mixing costs (Rubin and Meiran, 2005). Hence, the overlapping response-stimulus set seems to be a critical boundary condition that leads to observable differences in mixing costs between bilinguals and monolinguals (e.g., Mayr, 2001).

Importantly, the type of response mapping may still affect switch costs via changes in the demands of task-set reconfiguration (Meiran, 2000; Kiesel et al., 2010). For instance, Meiran (2008) demonstrates that the adverse effect of overlapping response mapping on switch costs is sensitive to the manipulation of time variables (e.g., the cue-to-stimulus interval), which directly influences task-set reconfiguration. Meiran argues that when the task cue switches from color to shape (or vice versa), the same response key in tasks with response overlapping should be recoded to match different aspects of the stimulus in a fraction of a second (i.e., response recoding), and this demands greater task-set reconfiguration. In contrast, response recoding is unnecessary in tasks with non-overlapping response mapping, in which each response is assigned to a separate key and therefore imposes smaller demands on task-set reconfiguration. Given that different types of response mapping may trigger divergent outcomes, future studies should closely examine the potential impact of task demands, which may modulate bilingual effects on task switching (e.g., Qu et al., 2015).

### Participant age

In view of the importance of varying task demand, it is plausible that an interplay between the participant's age and task demand moderates bilingual advantages in task switching. Since behavioral variability typically has a U-shaped function over the lifespan, with larger variability in children and older adults (Grady, 2012), task demand should be sensitive to capture performance differences in children and older adults, who are markedly different from young adults in terms of (a) cognitive maturation and deterioration and (b) extent of involvement in challenging cognitive activities other than language switching (Valian, 2015). Therefore, it is plausible that even a mildly difficult task should be sensitive to capture larger variability associated with bilingualism in the child or aging population. Given this, it is not surprising that the majority of studies that tested young adults—who have likely reached the peak of their cognitive functioning—have reported either null or inconsistent findings, primarily because testing young adults on tasks with mild or moderate difficulty appears to constrain potential variability between monolinguals and bilinguals (Bialystok, 2006; Costa et al., 2008).

In contrast, studies that tested either young children or older adults have reported relatively consistent bilingual advantages (Soveri et al., 2011; Gold et al., 2013; Houtzager et al., 2015). For instance, older bilinguals have shown smaller mixing costs than their monolingual counterparts (e.g., Gold et al., 2013). Likewise, bilingual children produced significantly lower post-switch errors–which are an equivalent index of task-switching performance–on a simple DCCS task (see Table 2; Bialystok, 1999; Bialystok and Martin, 2004; Carlson and Meltzoff, 2008). Taken together, these studies demonstrate that the bilingual's age warrants further consideration as a key factor in the developmental influence of bilingualism on task switching (i.e., shifting function).

**Table 2:** **Summary of studies examining bilingual advantages in task-switching among young children**.

**Study**	**Participants**	**Mean age**	**Task**	**Number of trials**	**Task-switching outcome**
Bialystok, 1999	Bilinguals (*N* = 30) Monolinguals (*N* = 30)	4.9	DCCS	20 trials (10 pre-switch trials and 10 post-switch trials)	Post-switch accuracy advantages in bilinguals
Bialystok and Martin, 2004	Chinese-English bilinguals (*N* = 31)	4.9	Computerized DCCS	80 trials (20 non-switch trials, 30 pre-switch trials, and 30 post-switch trials)	Post-switch accuracy advantages in bilinguals
	English monolinguals (*N* = 36)				
Carlson and Meltzoff, 2008	Spanish-English bilinguals (*N* = 12)	6.0	Advanced DCCS	20 trials (16 shape trials and 4 color trials)	Post-switch accuracy advantages in bilinguals
	Language immersion children (*N* = 21)				
	Monolinguals (*N* = 17)				
Barac and Bialystok, 2012	Chinese-English bilinguals (*N* = 30)	6.1	Color-shape task switching	200 trials (50 pure-repeat trials, and 150 trials with 50% of switch and 50% of repeat)	Mixing-cost advantages in all bilingual groups
	French-English bilinguals (*N* = 28)				
	Spanish-English bilinguals (*N* = 20)				
	English monolingual (*N* = 26)				

DCCS = Dimension Change Card Sort task.

## Conclusions

Given discrepancies in the literature, we propose that bilinguals' language-switching practices should be examined in ways that reflect the challenging quality of language control. Our review suggests that bilinguals' complex language-switching experiences (e.g., a dual-language context or frequent use of more demanding code-switching), task-specific demands on control processing, and potential moderators (e.g., age) will yield valuable insights on the link between bilingual experience and specific aspects of EF.

## Author contributions

HY, AH conceptualized the paper and HY, AH, and SY wrote the main paper and equally contributed to critical revision of this paper.

### Conflict of interest statement

The authors declare that the research was conducted in the absence of any commercial or financial relationships that could be construed as a potential conflict of interest.
